# Dr. Sweatman

**Published:** 1840-01

**Authors:** 


### DR. SWEATMAN.

At his house, in Berners-street, on the 18th September, Dr. Sweatman, Lecturer on Midwifery at the Middlesex Hospital, aged thirty-nine. Dr. Sweatman was deservedly respected for his abilities, and beloved for his moral worth.

